# Case report of antifungal drug-induced phlebitis in a patient with myelodysplastic syndrome

**DOI:** 10.1097/MD.0000000000040971

**Published:** 2024-12-20

**Authors:** Jia-Quan Wen, Xing-Yu Guo, Yan-Qun Xie, Jun-Jun Zhang, Jun-Hui Mei

**Affiliations:** a Department of Hematology and Oncology, International Cancer Center, Shenzhen Key Laboratory, Shenzhen University General Hospital, Shenzhen University Clinical Medical Academy, Shenzhen University Health Science Center, Shenzhen, China.

**Keywords:** amphotericin B cholesterol sulfate complex, case report^ine^, infrared therapy, myelodysplastic syndrome, phlebitis

## Abstract

**Rationale:**

This article summarized the nursing experience of phlebitis caused by intravenous infusion of amphotericin B cholesterol sulfate complex in a patient with myelodysplastic syndrome.

**Patient concerns:**

A 59-year-old man with myelodysplastic syndrome complicated with fungal infection was treated with antifungal therapy.

**Diagnoses:**

Patient with myelodysplastic syndrome was diagnosed with secondary phlebitis caused by liposomal amphotericin B.

**Interventions:**

The focus of nursing care was to use infrared rays of a specific electromagnetic wave therapy device to treat the patient’s phlebitis.

**Outcomes:**

After 30 minutes each time, twice a day, the patient’s phlebitis was cured after 6 days of continuous use.

**Lessons:**

The specific electromagnetic wave therapy can make the phlebitis caused by amphotericin B quickly heal, which is helpful for the smooth implementation of the antifungal treatment program.

## 1. Introduction

Myelodysplastic syndrome (MDS) is a heterogeneous clonal disorder of hematopoietic stem cells caused by the combined effects of genetic variations, malnutrition, or chemical agents. Its main features include ineffective hematopoiesis, bone marrow dysplasia, and peripheral blood cytopenias. As the disease progresses, it can lead to bone marrow failure and ultimately progress to acute myeloid leukemia.^[1]^ Chemotherapy is a common treatment modality for MDS at present. Especially for high-risk patients, it can effectively alleviate symptoms, prolong survival, and improve quality of life. However, chemotherapy can also exacerbate immune dysfunction, significantly increasing the risk of infections in MDS patients. Fungal infections are common complications of MDS chemotherapy, with an incidence rate of approximately 2.1% and a high mortality rate of 11.7% among patients undergoing chemotherapy, and their proportion in hospital-acquired infections has been rapidly increasing in recent years.^[2]^

With the increasing number of MDS patients undergoing chemotherapy, the treatment of fungal infections has become an important part of clinical management. Research has shown^[3]^ that amphotericin B is currently the preferred antifungal agent due to its low cost and broad spectrum of activity. The clinical overall response rate of intravenous amphotericin B is 67.3%, with a fungal clearance rate of 66.7%. Moreover, amphotericin B can reduce the costs of antifungal drugs, hospitalization, and disease management, possibly because of its high success rate as a first-line treatment, which reduces the need for second-line antifungal drugs, additional tests, prolonged hospital stays, and associated nursing costs and bed charges. Additionally, amphotericin B does not require dosage adjustments or changes in frequency when used in special populations such as the elderly or those with impaired renal function, providing greater clinical convenience and reducing the burden of care while achieving additional clinical benefits.

However, oral and intramuscular administration of amphotericin B is poorly absorbed and highly irritating. Therefore, peripheral intravenous infusion of amphotericin B is mainly used, but its prolonged use can lead to strong vascular irritation and the occurrence of phlebitis. Phlebitis is characterized by pain, redness, tenderness, and swelling at the infusion site. Failure to promptly intervene with effective measures can interrupt the course of amphotericin B therapy, affecting the treatment outcomes of patients. This not only increases patient suffering and economic burden but also may lead to thrombosis or even sepsis and death.^[4]^

Infrared therapy has shown good clinical value in the management of phlebitis, reducing patient pain while increasing comfort. Far-infrared therapy devices integrate far-infrared, electrical, and magnetic therapies into a single physical instrument, exerting photobiological effects by applying far-red light to the body, enhancing local cellular metabolism, and promoting blood circulation.^[5]^

In this study, we report a case of phlebitis induced by peripheral intravenous infusion of amphotericin B cholesterol sulfate complex in a patient with myelodysplastic syndrome. In our department, this case of phlebitis was treated with infrared therapy, resulting in rapid resolution of the phlebitis and successful implementation of the antifungal treatment regimen.

## 2. Case review

The patient, a 59-year-old male, was admitted to the hospital on February 19, 2024, complaining of “fever, chest tightness, and chest pain for 17 days, with a history of decreased whole blood cells for 16 days,” and reported dry and hard stools with a weight loss of 6 kg.

## 3. Present medical history

The patient had been diagnosed with myelodysplastic syndrome for 3 months and had been receiving regular chemotherapy. He developed fever without apparent cause on February 2, 2024, with a temperature of 39.6 °C, accompanied by fatigue, chest tightness, and chest pain aggravated after activity and during exhalation. On February 3, 2024, a chest computed tomography (CT) scan at an external hospital showed bilateral pneumonia, bilateral pleural effusion with partial compressive atelectasis of both lower lungs, and a small amount of pericardial effusion. During treatment at the external hospital, the patient developed septic shock and improved after fluid resuscitation, pressor support, and other treatments (specific treatment details unknown). A follow-up chest CT scan on February 13, 2024, showed increased pneumonia in the right middle lung compared to before, with decreased inflammation in the remaining lungs and bilateral pleural effusion, and slightly increased pericardial effusion compared to before. The diagnosis of myelodysplastic syndrome was confirmed on February 15, 2024, in combination with bone marrow biopsy results. After admission, the patient received empirical antifungal therapy with voriconazole. On February 21, 2024, the hypersensitive C-reactive protein was 260.28 mg/L. From February 20 to February 26, 2024, the antibiotics were adjusted by physicians to meropenem plus vancomycin plus voriconazole for combination antimicrobial therapy due to poor antimicrobial efficacy. On February 26, 2024, a follow-up chest CT scan showed progression of lung infection and pleural effusion compared to before, with a high possibility of fungal breakthrough. On February 27, 2024, the patient received additional liposomal amphotericin B complex for antifungal therapy. The first group received 500 mL of 5% glucose injection plus 100 mg of liposomal amphotericin B complex for injection, once daily for 18 days (February 27 to March 15, 2024), administered via a peripheral intravenous infusion pump (initial infusion rate of 20 mL/h for 0 to 2 hours, 30 mL/h for 3 to 4 hours, and 60 mL/h thereafter, with a total infusion time of more than 6 hours). The second group received 500 mL of 5% glucose injection plus 100 mg of liposomal amphotericin B complex for injection, once daily for 18 days (February 27 to March 15, 2024), at a constant infusion rate of 60 mL/h. The total duration of antifungal infection treatment was up to 18 days. To prevent infusion blockage, nurses flushed the catheter with 20 mL of 5% glucose injection every 2 hours and replaced the indwelling needle regularly. On March 9, 2024, the patient developed redness and swelling at the site of the previous puncture on both hands, measuring 3 × 4 cm, with tenderness and increased skin temperature. The patient experienced pain at the site, and was administered intramuscular injection of 50 mg tramadol hydrochloride injection for pain relief as per physician’s instructions. It was diagnosed with secondary phlebitis according to the “Nursing Treatment Practice Standards” (8th edition) issued by the Infusion Nurses Society in America.^[6]^ Nurses subsequently applied mupirocin ointment, lidocaine cream, and magnesium sulfate wet compress to the affected area, with unsatisfactory results. Infrared therapy was initiated by nurses on March 13, 2024, and continued for 6 consecutive days. On March 19, 2024, the patient’s phlebitis was cured.

## 4. Specific operations

1. Preheat the specific electromagnetic wave therapy device^[7]^ (Chongqing Xinfeng Medical Equipment Co., Ltd., 250 W, 50 Hz) for 5 minutes after turning it on.
2. Position the patient comfortably in a supine position, exposing either the left or right affected area at a time.
3. Adjust the irradiation distance so that the affected area is 20 cm vertically from the irradiation panel, with the closest distance based on the patient’s tolerance.
4. Irradiation time: 30 minutes per session, twice daily.
5. Check the patient every 15 minutes to prevent skin burns.

## 5. Discussion

For patients with hematologic malignancies, phlebitis can not only lead to sepsis, affecting the diagnosis and treatment of the disease, but also prolong hospitalization, increase the economic burden on patients, and add pressure to caregivers. It even increases the difficulty of venipuncture and nursing workload for healthcare workers. Therefore, finding effective ways to prevent and treat phlebitis is crucial.

Phlebitis^[6]^ is caused by various factors stimulating the vascular endothelium, resulting in inflammation of the vessel wall, local venous pain, redness, swelling, and, in severe cases, formation of inflammatory changes such as cord-like veins. The standard divides phlebitis into 4 levels: grade 0, no symptoms; grade 1, redness at the infusion site with or without pain; grade 2, pain at the infusion site with redness or swelling; grade 3, pain at the infusion site with redness or swelling, cord-like formation visible, and grade 4, pain at the infusion site with redness or swelling, visible cord-like veins >2.5 cm, and purulent discharge.

Research^[8]^ has pointed out the mechanism of non-chemotherapeutic drug-induced phlebitis: (1) High drug concentration, rapid infusion rate, exceeding the vascular buffering capacity, or drug accumulation at vascular damage sites can stimulate the vascular endothelium and cause phlebitis. (2) The insoluble particles in drugs, mainly including drug crystals and nondrug particles, are related to the occurrence of phlebitis. Insoluble particles account for about 70% of the occurrence of phlebitis. Insoluble particles stimulate the vascular endothelium during the process of blood circulation, leading to changes in the normal state of the vessel wall and endothelial damage, which triggers platelet aggregation and adhesion, thereby inducing phlebitis.

Peripheral intravenous infusion of liposomal amphotericin B is highly irritating to blood vessels, with long infusion times, high concentrations, and a high likelihood of causing phlebitis. In this case, liposomal amphotericin B cholesterol sulfate complex was used. When preparing the drug, strict adherence to aseptic and on-demand requirements was maintained. According to the instructions, sterile water for injection was used to dissolve the drug, and 5% glucose injection volume recommended by the manufacturer was rapidly added to the bottle, which was gently shaken and rotated to ensure complete dissolution. During infusion, the drug was protected from light, and a separate light-resistant infusion pump was used to minimize particle contamination. Following the medication guidance^[9]^ and physician’s instructions, the infusion time for the first group of liposomal amphotericin B cholesterol sulfate complex exceeded 10 hours, and for the second group, it exceeded 8 hours, with a total infusion time of approximately 20 hours. Although 20 mL of 5% glucose injection was used to flush the catheter every 2 hours, and the indwelling needle was regularly replaced during this period, the long infusion time and duration of treatment reached 18 days. Additionally, the drug concentration of liposomal amphotericin B is positively correlated with the occurrence of phlebitis. Studies have shown that the incidence of phlebitis significantly decreases after reducing the drug concentration. In this case, according to the recommended dose of 3.0 to 4.0 mg/kg in the instructions, the dose of liposomal amphotericin B cholesterol sulfate complex for each group reached 100 mg. Although our department used a microinfusion pump for slow intravenous infusion and strictly controlled the infusion rate, the maximum drug concentration reached 12 mg/h. Therefore, the occurrence of phlebitis is not surprising. According to the phlebitis grading criteria, the phlebitis in this case was classified as grade II. Recommended treatment methods in the “Nursing Treatment Practice Standards” include local cold compress with ice packs for 1 to 2 hours, which reduces local temperature, gradually contracts blood vessels, relieves bleeding, bruising, and swelling, and decreases metabolism and nerve conduction to achieve anti-inflammatory and analgesic effects. Another method is to moisten gauze pads with 50% magnesium sulfate solution. Magnesium sulfate is a hypertonic solution, and both Mg^2+^ and SO_4_²⁻ are strong polar substances. Both can absorb water from tissues due to concentration differences, thereby reducing swelling, and so on. After the occurrence of this case, our department applied mupirocin ointment, lidocaine cream, and magnesium sulfate wet compress successively, with unsatisfactory results and even a trend towards worsening. Our department chose to use a specific electromagnetic wave therapy device for infrared therapy, and after 6 consecutive days of treatment, the patient’s phlebitis was cured.

The specific electromagnetic wave therapy device is a 3-in-1 therapeutic instrument integrating magnetic therapy and infrared therapy. Through the irradiation of far-infrared wavelengths and magnetic therapy, the human body’s metabolism can be promoted. The basis of infrared therapy lies in the thermal effect. Infrared radiation has strong penetration through human skin and subcutaneous tissue. Under infrared irradiation, tissues heat up, generating a thermal effect that promotes blood circulation and metabolism, increases cellular phagocytic function, eliminates swelling, and has anti-inflammatory and regenerative effects.^[10]^ Additionally, far-infrared wavelengths are close to the vibration frequency of human cell molecules. After irradiation on the human body, some cell atoms and molecules resonate, generating heat through molecular friction, increasing local temperature, dilating local blood vessels, improving blood circulation, facilitating the removal of harmful substances and vascular deposits, thereby reducing local pain and swelling.^[5]^

## 6. Conclusion

In summary, the specific electromagnetic wave therapy can make the phlebitis caused by amphotericin B quickly heal, which is helpful for the smooth implementation of antifungal treatment program. Timely and effective intervention for phlebitis is crucial for patient recovery and health, as it can alleviate symptoms, prevent complications, improve treatment outcomes, promote wound healing, prevent recurrence, and ensure patient health and quality of life. The nursing team should closely monitor the patient’s condition, understand the mechanism of phlebitis, identify and diagnose early phlebitis to ensure effective and rapid treatment.

## Acknowledgments

This patient with antifungal drug-induced phlebitis in Department of Hematology and Oncology, and Shenzhen University General Hospital, are appreciatively acknowledged.

## Author contributions

**Data curation:** Jia-Quan Wen, Xing-Yu Guo.

**Investigation:** Xing-Yu Guo, Jun-Jun Zhang.

**Project administration:** Jun-Hui Mei.

**Resources:** Jia-Quan Wen, Xing-Yu Guo, Yan-Qun Xie.

**Software:** Jia-Quan Wen.

**Writing – original draft:** Jun-Jun Zhang.
